# Exploring Sodium Glucose Co-Transporter-2 (SGLT2) Inhibitors for Organ Protection in COVID-19

**DOI:** 10.3390/jcm9072030

**Published:** 2020-06-28

**Authors:** Beatriz Fernandez-Fernandez, Luis D’Marco, Jose Luis Górriz, Conxita Jacobs-Cachá, Mehmet Kanbay, Sergio Luis-Lima, Esteban Porrini, Pantelis Sarafidis, María José Soler, Alberto Ortiz

**Affiliations:** 1 IIS-Fundación Jiménez Diaz, Department of Medicine, School of Medicine, Universidad Autónoma de Madrid, 28029 Madrid, Spain; BFernandez@fjd.es (B.F.-F.); sergio.lima@quironsalud.es (S.L.-L.); 2 Red de Investigación Renal (REDINREN), Instituto Carlos III-FEDER, 28040 Madrid, Spain; conxita.jacobs@vhir.org (C.J.-C.); esteban.l.porrini@gmail.com (E.P.); m.soler@vhebron.net (M.J.S.); 3 Department of Nephrology, Hospital Clínico Universitario, INCLIVA, 46010 Valencia, Spain; luisgerardodg@hotmail.com (L.D.); jlgorriz@gmail.com (J.L.G.); 4 Medicine Department, Universidad de Valencia, 46010 Valencia, Spain; 5 Nephrology Department, Hospital Universitari Vall d’Hebron, Universitat Autònoma de Barcelona, Nephrology Research Group, Vall d’Hebron Research Institute, 08035 Barcelona, Spain; 6 Department of Medicine, Division of Nephrology, Koc University School of Medicine, 43010 Istanbul, Turkey; drkanbay@yahoo.com; 7 Department of Medicine, Hospital Universitario de Canarias, 38320 Tenerife, Spain; 8 Instituto de Tecnologías Biomédicas, University of La Laguna, 38320 Tenerife, Spain; 9 Department of Nephrology, Hippokration Hospital, Aristotle University of Thessaloniki, 54642 Thessaloniki, Greece; psarafidis11@yahoo.gr

**Keywords:** COVID-19, diabetes, chronic kidney disease, cardiovascular, SGLT2, canagliflozin, dapagliflozin, empagliflozin

## Abstract

Hospital admissions and mortality from the Coronavirus disease 2019 (COVID-19) pandemic are spreading throughout the world, and second and third waves are thought to be likely. Risk factors for severe COVID-19 include diabetes, chronic kidney disease and cardiovascular disease. Currently, there is no vaccine and no approved therapy. Therapeutic approaches are aimed at preventing viral replication and spread, limiting the impact of the inflammatory overdrive (cytokine storm), preventing thromboembolic complications and replacing or supporting organ function. However, despite organ support, mortality is currently 65% for those receiving advanced respiratory support and 78% for those requiring renal replacement therapies. Thus, efforts should be made to provide adjuvant organ protection therapy. This may imply novel therapies in clinical development (e.g., the Fas ligand trap asunercept), but uptake of repurposed drugs already in clinical use may be faster. In this regard, sodium glucose co-transporter-2 (SGLT2) inhibitors were recently shown to protect the heart and kidney both within and outside of a diabetic milieu context. Further, preclinical data support a beneficial effect for the lung. We now discuss the potential benefits and risks of SGLT2 inhibitors in COVID-19 and an ongoing clinical trial testing the impact of dapagliflozin on outcomes in COVID-19 patients with respiratory failure.

## 1. Introduction

Coronavirus disease 2019 (COVID-19) is a pandemic caused by the severe acute respiratory syndrome (SARS)-Coronavirus (CoV)-2. This is the third recent outbreak of coronaviruses following the SARS-CoV (2002) and the Middle East respiratory syndrome (MERS)-CoV (2015). It originated from the Chinese city of Wuhan in December 2019 and as of 8 June 2020 has infected more than 7 million people worldwide with more than 400,000 deaths [1,2,3]. Most common presenting symptoms include fatigue, fever, cough, dyspnea and diarrhea while median time between symptoms onset and death is 14 days [4,5,6]. The mortality rate of 2.8% (median age of 75 years) is lower than the 10% mortality in SARS-CoV and 37% mortality in MERS-CoV; however, higher absolute numbers of cases and deaths are explained by easy transmission via direct contact or droplets, as well as transmission by asymptomatic or mildly symptomatic individuals [4,7,8,9]. Although the search of reservoir animals has been inconclusive so far, there is 88% genetic similarity between SARS-CoV-2 and two bat-derived coronaviruses [10,11,12]. Possible fecal-oral transmission has also been reported while vaginal virus delivery has not been proven [13,14,15].

The estimated mortality of diagnosed cases ranges from 2.3% to 15.2%, depending on patient populations, diagnostic strategies and, probably, additional poorly characterized factors [16,17]. The need for intensive care unit (ICU) admission is around 20% [18]. In ICUs, about 90% require respiratory support via either mechanical (88%) or non-invasive ventilation (12%) and mortality hovers around 50% [19]. Older age, higher sequential organ failure assessment (SOFA) score, D-dimer >1 μg/mL, presence of comorbid diseases or secondary infections, elevated inflammatory markers, low CD3+ and CD8+ T cell counts and cardiac troponin >0.05 ng/mL predict mortality while acute respiratory failure and fulminant myocarditis are the most commonly reported causes of death [20,21,22].

Herein, we now review the current status of therapy for COVID-19 emphasizing the concept of organ protection and the potential beneficial role of sodium glucose co-transporter-2 (SGLT2) inhibitors in the COVID-19 context.

## 2. Risk Factors for Severe COVID-19

Clinical conditions associated with a higher risk for severe complications and death in COVID-19 infected patients include cardiovascular disease, diabetes and chronic kidney disease (CKD). A recent meta-analysis including 1527 patients from six different studies, mostly from Wuhan (China), showed that hypertension (17.1%), cardiovascular disease (16.4%), and diabetes (9.7%) were the most prevalent diseases observed in patients with severe COVID-19 complications [23]. The fatality rate was 10.5, 7.3 and 6% in persons with cardiovascular disease, diabetes and hypertension, respectively, whereas the overall fatality rate was 2.3% in the COVID-19 summary report of 72,314 cases released by the Chinese Center for Disease Control [24]. Thus, patients with previous cardiovascular disease and diabetes had more severe disease and more complications [25]. Cardiovascular disease and diabetes are closely linked to CKD. In this regard, a meta-analysis of four studies, which included 1389 COVID-19 patients, disclosed that CKD was associated with a severe presentation of the disease (OR, 3.03; 95%CI, 1.09–8.47), despite none of the individual studies showing this association [26]. The likely explanation may be the higher number of patients analyzed in the meta-analysis. COVID-19 may also be complicated by acute kidney injury (AKI), which developed in 5.1% of 701 adults in a prospective cohort study [27]. Mortality was higher in patients with elevated than normal serum creatinine at baseline: HR, 2.10; 95%CI, 1.36–3.26. Additionally, although not prominent in early Wuhan reports, renal disease was the most common comorbidity among the first 6720 reported critically ill COVID-19 patients in the UK, being five-fold more common than cardiovascular disease [28].

## 3. Current Therapeutic Approaches to COVID-19

**Figure 1:**
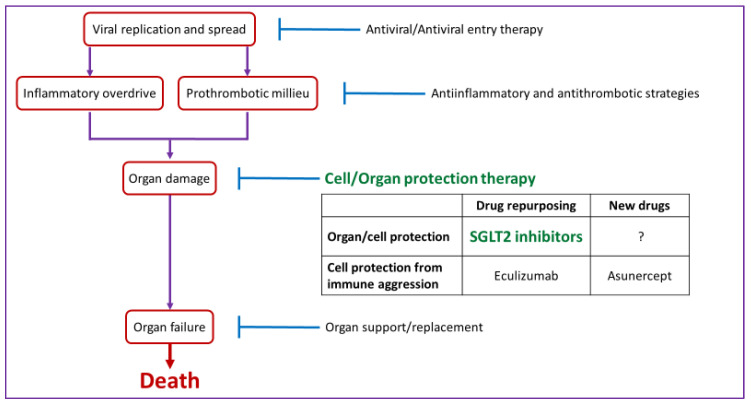
Pathogenic basis of current therapeutic approaches to COVID-19 and potential place of SGLT2 inhibitors.

There is no specific treatment for SARS-CoV-2. Several studies aiming to identify novel therapeutic agents and vaccines against SARS-CoV-2 are ongoing, but their safety and efficacy need to be assessed before generalized use [29,30,31]. In this emergency context, the health community has initially promoted the off-label use of approved drugs to try to control the SARS-CoV-2 infection as well as to manage the inflammatory, thrombotic, respiratory and cardiovascular complications in critically ill patients (Figure 1). However, therapies aimed at organ protection have lagged behind.

### 3.1. Antiviral Therapies

**Figure 2:**
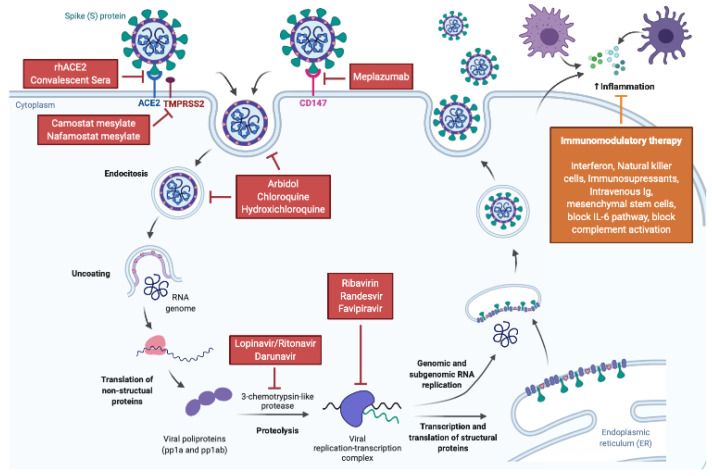
Scheme of the SARS-CoV-2 replication cycle, the host immune response and potential drug targets. This picture shows a simplified SARS-CoV-2 life cycle in the target cells as well as a representation of the immune response against the virus. The targets of antiviral therapies and immunomodulation approaches are highlighted.

The primary goal during the initial phases of SARS-CoV-2 infection is to stop the virus cell cycle and slow down disease progression. The SARS-CoV-2 cell cycle resembles that of other coronaviruses (i.e., SARS-CoV and MERS-CoV) and shares replication mechanisms with other RNA viruses (i.e., HIV or Ebola). Therefore, approved drugs to treat other viral infections or with a potential to interfere in viral propagation mechanism have been tested in COVID-19 patients [29,30,32,33]. Coronaviruses target host cells via direct interaction of the viral spike (S) protein with a cell membrane protein. In particular, SARS-CoV-2, like SARS-CoV, enters host cells via the angiotensin converting enzyme 2 (ACE2), an endocytosis process facilitated by the type 2 transmembrane serine protease (TMPRSS2) [34,35]. A recent preprint report described SARS-CoV-2 entry into host cells by interacting with basigin, also known as extracellular matrix metalloproteinase inducer (EMMPRIN) or cluster of differentiation 147 (CD147) [34]. Once inside cells, viruses promote the translation of their non-essential polyproteins that after proteolysis will form a replication-transcription complex to replicate the genomic RNA and produce viral proteins. Then, new viral particles are assembled and released, starting the cycle again (Figure 2). 

The virus-host cell recognition may be prevented by soluble recombinant human ACE2 (rhACE2) or convalescent SARS-CoV-2 sera [36,37]. The potential therapeutic role of rhACE2 for COVID-19 is under study as it decreased SARS-CoV-2 infectivity in human engineered blood vessels and kidney organoids [38,39]. Blocking CD147-mediated virus entry could also be a therapeutic option [40]. Meplazumab, a humanized antibody against CD147, was reported to accelerate COVID-19 resolution in seventeen COVID-19 patients [41]. In addition, viral endocytosis can be inhibited by drugs. Camostat mesylate and nafamostat mesylate are serine protease inhibitors that target TMPRSS2 and block SARS-CoV-2 infection in human lung cells [35,42]. Camostat mesylate is approved in Japan to treat pancreatitis and COVID-19 clinical trials are ongoing [43]. Chloroquine and its hydroxylated form (hydroxychloroquine) were discovered in the early 30′s and 50′s, respectively. Both drugs are weak bases that accumulate in acidic compartments such as lysosomes and autosomes, interfering with normal endocytosis processes. Chloroquine was initially developed to treat malaria and hydroxychloroquine to treat rheumatoid arthritis, but they are now approved to treat diseases ranging from cancer to chronic inflammatory diseases [44]. Because of a known safety profile and low cost, they are being tested for both COVID-19 prevention and treatment with encouraging results although their efficacy has been questioned [33,45,46]. Arbidol (umifenovir) is a broad-spectrum antiviral drug that blocks the virus-cell membrane fusion by intercalating into membrane lipids [47,48]. Its use is approved for influenza treatment in Russia and China, and it is being tested in clinical trials for COVID-19. Wuhan data report better outcomes in patients treated with arbidol alone or in combination with other antiretroviral drugs [49,50]. Drugs that block viral RNA replication include lopinavir/ritonavir and darunavir, which inhibit the viral 3-chymotrypsin-like protease and are second line antiretrovirals for HIV [51]. However, the first randomized clinical trial (RCT) testing Lopinavir/Ritonavir suggested a limited efficacy for SARS-CoV-2 infections [52]. Ribavirin, remdesivir and favipiravir are broad-range viral RNA polymerase blockers that have been previously tested for Hepatitis C, Ebola or Influenza, among others [53,54,55,56,57]. Their efficacy in COVID-19 is currently being tested in several RCTs following the publication of case reports of successful remdesivir or favipiravir use [58,59,60].

### 3.2. Anti-Inflammatory Therapies

In later disease stages, immunomodulation strategies should be considered. Viral infections are met by a regulated immune response aimed at fighting the virus. However, excessive release of proinflammatory cytokines and chemokines may lead to a “cytokine storm”, thought to be the main cause of the SARS-CoV-2 acute respiratory distress syndrome, which is associated to thromboembolic events leading to multi-organ failure and death [61,62]. Although immunomodulation therapies remain controversial in the COVID-19 context because they may negatively influence the natural immune response against the virus [63], several ongoing RCTs aim to minimize immune system over-activation [30]. The main immunomodulatory strategies are [29] enhancing innate immune responses by administering recombinant interferon or natural-killer cells and [30] decrease inflammatory responses via the use of corticosteroids and other immunosuppressants, intravenous administration of general or SARS-CoV-2-specific immunoglobulins, infusion of mesenchymal stem cells, and blocking interleukin-1 (anakinra), interleukin-6 (tocilizumab, sarilumab) or complement activation (eculizumab and 5 additional agents are in clinical trials) [30,64]. Finally, it has been suggested that extracorporeal treatment, for example high-volume hemofiltration, may play a role in patients with COVID 19 and cytokine storm [65].

### 3.3. Anti-Thrombotic Strategies

Classical anticoagulant therapies were initially applied to COVID-19 patients at risk of thrombotic and thromboembolic events [66]. However, since COVID-19 patients with acute respiratory failure present severe hypercoagulability with fibrin formation and polymerization that may predispose to thrombosis and correlates with worse outcome, the use of low molecular weight heparin was extended to patients not requiring admission, especially if there is a high risk of venous thromboembolism (e.g., reduced mobility or co-morbidities), elevated D-dimer (>2 times the upper limit normal) and low risk of bleeding [67].

### 3.4. Organ Support, Replacement and Protection

In some cases, organ failure needs organ replacement or support therapies. The most common are respiratory support (advanced respiratory support was required by 70% of UK critically ill patients), renal replacement therapies (required in 22% of these patients) and cardiovascular support [68]. Mortality was 65% for those receiving advanced respiratory support and 78% for those requiring renal replacement therapies. The high mortality of patients requiring organ support points to the need to develop and implement organ protective strategies. As an example, Apogenix is to start clinical trials of asunercept (APG101) for COVID-19. This is, a human fusion protein consisting of the extracellular domain of the CD95 (Fas, TNFRSF6) receptor and the Fc domain of an IgG antibody that prevents Fas-induced epithelial cell death. Fas is upregulated in kidney cells during systemic inflammation and promotes lung and kidney cell death [69,70]. However, despite potential to protect both lung and kidney, asunercept is still in early stages of clinical development [71]. Drug repurposing offers the possibility of accelerated clinical translation. In this regard, SGLT2 inhibitors were recently shown to have heart and kidney organ protective properties both within and outside of a diabetic milieu context [72,73,74] and could offer an opportunity for organ protection in COVID-19.

### 3.5. SGLT2 Inhibitors: From Glucose Lowering to Organ Protection

SGLT2 inhibitors are oral hypoglycemic agents for patients with type 2 diabetes mellitus (Τ2DM) [75]. One member of the class, dapagliflozin, was also recently approved in Europe for patients with T1DM. They block SGLT2, a high-capacity/low-affinity glucose transporter located in the S1 segment of renal proximal tubules and responsible for 90% of glucose reabsorption [76]. In diabetic patients, SGLT2 expression increases up to 3-fold. However, this effort to preserve glucose by preventing urinary excretion further deranges glucose homeostasis. SGLT2 inhibition results in excretion of 50-60% of filtered glucose, roughly corresponding to 60–100 g/day and loss of the associated calories [76,77]. 

SGLT2 inhibitors have beneficial metabolic and cardiovascular effects. Loss of glucose in urine results in weight loss of around 2–4 kg within 3–6 months [78]. Further, the double mild natriuretic action (SGLT2 inhibition and the osmotic effect of glucosuria) causes clinically meaningful blood pressure reductions of 4–5/2–3 mmHg [79]. SGLT2 inhibition decrease of sodium reabsorption results in decreased energy expenditure by proximal tubular cells, potentially influencing other functions of these metabolically active cells. SGLT2 inhibitors are generally well-tolerated, with the most frequent adverse event being genital mycotic infections and the most serious the rare development of euglycemic diabetic ketoacidosis (DKA) [80].

Accumulating evidence suggest that SGLT2 inhibitors are potent cardioprotective and nephroprotective agents [81]. The Empagliflozin Cardiovascular Outcome Event Trial in Type 2 Diabetes Mellitus Patients (EMPA-REG OUTCOME), which randomized 7020 patients with T2DM and established cardiovascular disease to empagliflozin or placebo, first showed reductions of 14% in the primary outcome (non-fatal myocardial infarction or stroke, or death from cardiovascular causes) (HR, 0.86; 95%CI, 0.74–0.99; *p* = 0.04 for superiority), 38% in cardiovascular death, 35% in hospitalization for heart failure (HHF) and 32% in all-cause mortality [82]. The Canagliflozin-Cardiovascular-Assessment-Study (CANVAS) randomized 10,142 T2DM patients with either established cardiovascular disease or multiple cardiovascular risk factors to canagliflozin or placebo and also showed 14% reduction in the same primary outcome (HR, 0.86; 95%CI, 0.75–0.97); *p* = 0.02 for superiority), 33% reduction in HHF and a non-significant trend towards lower all-cause mortality (HR, 0.87; 95%CI, 0.74–1.01) [83]. The Multicenter Trial to Evaluate the Effect of Dapagliflozin on the Incidence of Cardiovascular Events Thrombolysis in Myocardial Infarction 58 (DECLARE-TIMI 58) trial included 17,160 patients (10,186 without atherosclerotic cardiovascular disease, i.e., a lower-risk population) and showed non-inferiority of dapagliflozin in the aforementioned primary composite outcome and superiority compared to placebo with regards to HHF (HR, 0.73; 95%CI, 0.61–0.88) [84]. Finally, the recent DAPAgliflozin in Heart Failure (DAPA-HF) study included 4744 patients (with or without T2DM) with NYHA II-IV heart failure and an ejection fraction <40% (heart failure with reduced ejection fraction) and showed that dapagliflozin significantly reduced the primary outcome of worsening heart failure or cardiovascular death (HR, 0.74; 95%CI, 0.65–0.85) in both diabetic and non-diabetic patients [73,85]. 

In parallel, preliminary evidence suggested that SGLT2 inhibitors significantly reduce albuminuria in comparison to placebo or active antihyperglycemic agents [86], while all the cardiovascular safety trials (EMPA-REG OUTCOME, CANVAS, DECLARE-TIMI) showed prominent effects on outcomes associated with kidney disease progression [83,84,87]. In a meta-analysis of these trials, SGLT2 inhibitors reduced the incidence of the composite renal outcome of worsening renal function (doubling of serum creatinine accompanied by an eGFR of ≤45 mL/min/1.73 m^2^), end-stage renal disease (ESRD) defined as eGFR <15 mL/min/1.73 m^2^) or renal death (defined as: “any adjudicated non-CV death event where the adjudication committee assigned a renal proximate cause is considered a renal death”) by 45% (HR, 0.55; 95%CI, 0.48–0.64) [88]. The recent CREDENCE study in 4401 patients with T2DM and diabetic kidney disease (CKD and albumin-to-creatinine-ratio 300–5000 mg/g) was prematurely stopped, showing reductions of 34% in the composite of ESRD, doubling of serum creatinine, or renal death and 32% in ESRD with canagliflozin compared to placebo [87]. Recently, the Study to Evaluate the Effect of Dapagliflozin on Renal Outcomes and Cardiovascular Mortality in Patients with Chronic Kidney Disease (DAPA-CKD), a renal outcome trial evaluating the effect of dapagliflozin in patients with diabetic or non-diabetic CKD was also prematurely stopped due to benefit [89].

The use of eGFR as an endpoint has been criticized since there is evidence that SGLT2 may reduce muscle mass and this may be associated with lower serum creatinine values [90]. However, this criticism does not apply to short term studies in which GFR was measured [91], as well as to albuminuria or hard (death) renal endpoints.

Several hypotheses on the mechanisms contributing to the cardioprotective properties of SGLT2 inhibitors were generated, including reductions in glycemia, body weight, visceral adiposity, uric acid, albuminuria, sympathetic tone, a shift from glucose to fatty acid oxidation resulting in increased levels of β-hydroxybutyrate which is a better cardiac fuel or a direct effect of SGLT2 inhibitors on cardiomyocytes [92,93]. However, the dissociation of the curves for the outcomes that displayed the larger differences between groups in these trials (HHF and cardiovascular death) were observed during the first weeks suggesting that hemodynamic mechanisms, including mild natriuresis and reductions in blood pressure, may play a more prominent role [81]. Similarly, anti-inflammatory and antifibrotic effects and reversal of renal hypoxia of these agents could help towards a renoprotective action [94,95,96], but modulation of renal microcirculation is the most possible renoprotective mechanism [81]. Briefly, DM promotes renal injury through afferent arteriole vasodilation resulting in increased intraglomerular pressure and albuminuria. SGLT2 inhibitors through inhibition of sodium reabsorption in proximal tubules increase its distal availability; this is sensed by the macula densa, which restores the tubuloglomerular feedback resulting in reversal of the vasodilation of the afferent arteriole, decreased intraglomerular pressure and proteinuria [81,97]. However, additional potential mechanisms of action remain underexplored. Thus, β-hydroxybutyrate modulates epigenetic regulation of gene expression by promoting histone Lysine β-hydroxybutyrylation, a histone mark that promotes the expression of cell protective genes such as Ppargc1a (which encodes PGC-1α) [97,98,99].

Based on the above findings, several consensus reports from major international bodies recommend SGLT2 inhibitors as the drug of choice after metformin in patients with T2DM and evidence of atherosclerotic cardiovascular disease, CKD or heart failure [81,100]. Additionally, clinical guidelines are starting to recommend SGLT2 inhibitors for patients with heart failure with reduced ejection fraction with or without diabetes [101,102].

More recently, preclinical studies, although limited to the vasculature, suggest a potential role for SGLT2 inhibitors in lung protection. Thus, in ex vivo studies, canagliflozin relaxed mouse pulmonary arteries in a dose-dependent manner [103]. In rat pulmonary arterial hypertension, empagliflozin improved survival, reduced medial wall thickening and decreased muscularization of pulmonary arterioles [104]. However, the molecular mechanisms of this protective effect remain unclear, despite the observation that empagliflozin was associated with increased apoptosis and decreased proliferation in pulmonary vessels and thus prevented adverse pulmonary arteriole remodeling.

Overall, there is accumulating clinical and preclinical data suggesting that SGLT2 inhibitors have the potential to provide organ protection both inside and outside of the diabetic context.

## 4. Current Recommendations and Potential Dangers of SGLT2 Inhibitors during Acute Illness

During acute illness, care should be taken with volume depletion, blood pressure reduction and DKA when prescribing SGLT2 inhibitors. In the context of COVID-19, there is additionally the potential for interaction with other drugs. In this regard, health agencies (Royal United Hospitals, NHS Foundation, Catalan Health agency, among others) have warned about safety concerns for SLGT2i and the risk of DKA in patients with both diabetes and COVID19 infection. In this setting, they have recommended to avoid SGLT2 inhibitors in all patients with risk factors for developing symptoms or serious complications of COVID-19, such as patients with a history of hypertension, T2DM, atherosclerotic cardiovascular disease, heart failure and/or estimated GFR <25 mL/min/1.73 m^2^. In this regard, COVID-19 patients may decrease food intake and may develop diarrhea and increased insensitive fluid losses through respiration and fever. Nevertheless, there is no published study to support this recommendation. It would probably be necessary to individualize the recommendation to discontinue SGLT2 inhibitors only in COVID-19 infected patients who present symptoms that predispose to a decrease in blood pressure or vascular volume, to avoid both the deterioration of renal function or DKA.

### 4.1. Volume Depletion

SGLT2 inhibitors may cause volume depletion leading to acute kidney injury, usually within well-defined circumstances such as concomitant use of high-dose loop diuretics, low baseline blood pressure, treatment with non-steroidal anti-inflammatory drugs or concomitant digestive problems impairment [86]. Moreover, the glomerular hemodynamic effects of combining renin-angiotensin system (RAS) blockade and SGLT2 inhibitors usually cause a mild decrease in glomerular filtration rate, which is associated with long-term preservation in renal function [105]. Nevertheless, meta-analyses comparing Canagliflozin or Dapagliflozin with either placebo or active comparator and one comparing Empagliflozin with placebo did not confirm a significant volume depletion with SGLT2 inhibitors [106]. In any case, hospitalized patients with mild-moderate manifestations of COVID-19 should be monitored to prevent volume depletion and renal impairment.

### 4.2. Blood Pressure

SGLT2 inhibitors tend to decrease blood pressure due to their osmotic diuretic effect, thus potentially causing orthostatic hypotension and dizziness, especially when combined with diuretics [107]. Other diuresis-independent actions leading to blood pressure reduction, including effects on ion transporters, sympathetic nervous system activity, and vascular function, have also been proposed, although clinical evidence supporting these is sparse.

### 4.3. Ketoacidosis

Regulators warn of the risk of DKA, especially in T1DM patients receiving SGLT2 inhibitors, 5% to 12% of whom develop DKA [107]. An increased risk of DKA was also recorded in T2DM RCTs of SGLT2 inhibitors [108,109]. In 44,000 patient-years, DKA incidence rates were 0.16–0.76 events per 1000 patient-years for an overall incidence of <0.1% of all treated patients [107], and in all cases, a precipitating factor was present. The risk factors for SGLT2 inhibitors-associated DKA are those conditions leading to restricted food intake or severe dehydration, alcohol abuse, sudden reduction in insulin or increased insulin requirements due to acute illness such as low beta cell function reserve (patients with T2DM who have low C-peptide levels, latent autoimmune diabetes in adults (LADA) or a history of pancreatitis) [108]. “Sick day rules” to manage diabetes during intercurrent illness help to prevent future complications [110]. In this regard, patients presenting COVID-19 symptoms may decrease food intake, blood pressure or vascular volume. SGLT2 inhibitors should be discontinued immediately if DKA is suspected or diagnosed since DKA requires urgent hospital admission. Physicians and patients should be aware of early symptoms associated to DKA (thirst, polyuria and sweet, fruity breath, abdominal pain and vomiting) in order to stop them timely.

### 4.4. Interactions with Experimental COVID-19 Drugs

Most interactions of experimental drugs currently used for COVID-19 are related to the potential increased or decreased exposures to or toxicities from co-medication therapies or COVID-19 drugs. In this regard, both hydroxychloroquine and azithromycin prolong the QT interval, and their association may trigger fatal torsade de pointes arrhythmia [111]. The currently known drug interactions can be consulted at https://www.covid19-druginteractions.org/. As of 28 April 2020, no expected interactions for empagliflozin or dapagliflozin were listed, while canagliflozin may potentially interact with lopinavir-ritonavir (Kaletra) requiring monitoring and eventual drug dosing modifications.

## 5. Clinical Trials of SGLT2 Inhibitors in COVID-19

Summarizing the current situation for COVID-19, there is no therapy approved for COVID-19, and current experimental therapies are aimed at preventing viral replication and spread, limiting the impact of the inflammatory overdrive (cytokine storm), preventing thromboembolic complications and replacing or supporting organ function. However, mortality in patients requiring organ support exceeds 60%. Thus, efforts should be made to provide adjuvant organ protection therapies. Despite a potential higher risk for SGLT2 inhibitors adverse effects in the context of acute illness and the resulting current advise against using them in COVID-19 patients, as recently emphasized [112], they have a favorable efficacy profile in preserving renal function, decreasing the impact of heart failure and increasing the survival of patients with risk factors for severe COVID-19. In preclinical studies, SGLT2 inhibitors had lung protective effects by decreasing oxidative stress, tissue hypoxia and inflammation [81,113,114,115]. Such an uncertainty regarding the use and risk-benefit balance of commonly prescribed drugs is not new in the history of treatment for severe disease or in the COVID-19 context. Beta blockers were contraindicated for many years in patients with heart failure, until RCTs showed benefit. Regarding COVID-19, many physicians rushed to stop RAS blockade in patients with suspected COVID-19 (personal observation) following a hypothesis that these drugs might be detrimental for COVID-19 as in animal models they increase ACE2 (the receptor of SARS-CoV-2) expression in cardiac [116] and vascular tissue [117]. The influence of RAS blockers on lung ACE2 expression is unknown both in experimental models and in humans. On the other hand, the protective effects of ACE2 have been widely described. Thus, an increased expression of ACE2 may be protective rather than detrimental in the COVID-19 context and rhACE2 is being pursued as a potential therapy for COVID-19 [118,119]. Clear statements by multiple scientific societies tried to dispel these doubts, supporting continuous use of RAS blockade, unless an established contraindication is met [120,121,122].

Given the uncertain risk-benefit balance of SGLT2 inhibitors, a clinical trial would be needed to address the issue. In this regard, from 15 April 2020, an ongoing RCT (Dapagliflozin in Respiratory Failure in Patients with COVID-19, DARE-19, NCT04350593) is testing dapagliflozin in patients with COVID-19 and respiratory failure to assess impact on disease progression, complications and all-cause mortality. In this international, multicenter, phase 3 double blind RCT, patients will initiate treatment with dapagliflozin 10 mg or placebo, once a day for 30 days on top of the standard local protocol for COVID19 treatment. The trial will randomize 900 hospitalized COVID-19 patients with mild-moderate disease severity, not needing mechanical ventilator support at the time of screening and having a prior cardiovascular or renal condition that impairs outcomes. Inclusion criteria comprise confirmed SARS-CoV-2 infection with chest radiography or CT scan compatible with COVID-19, mild-moderate disease (SpO2 ≥94% with ≤3 L supplemental oxygen) and at least one of the following conditions: hypertension, T2DM, atherosclerotic cardiovascular disease, heart failure and/or CKD stage 3 to 4 (eGFR 25–60 mL/min/1.73 m^2^). Key exclusion criteria include short life expectancy (<24 h), SBP <95 mm Hg and/or needing inotropic medication or mechanical circulatory support at screening, T1DM, treatment in the last 14 days or under treatment with anti-immunological drugs for COVID-19, DKA history in the prior 6 months or treatment with any SGLT2 inhibitors at screening or within the prior 4 weeks. The primary endpoint will be time to first occurrence of all-cause mortality or new/worsened organ dysfunction through 30 days of follow up. Organ dysfunction may consist of respiratory decompensation, heart failure, cardiovascular instability, ventricular tachycardia or fibrillation or need for drug or mechanical circulatory support or renal replacement therapy.

A second trial will assess the efficacy of the novel immunomodulatory agent EDP1815 (a strain of Prevotella histicola) and a combination of the approved cardiovascular drugs dapagliflozin and ambrisentan as potential treatments for COVID-19 disease against Standard of Care alone (NCT04393246).

A recent report of the off-label prescription of empagliflozin 10 mg for 5–7 days in three non-diabetic hospitalized patients with severe bilateral interstitial COVID-19-related pneumonia concluded that no favorable action related to treatment was obtained [123]. However, it is unclear how this conclusion may be reached in the absence of controls. The three patients apparently survived, which should be considered a good outcome for a potentially lethal disease.

## 6. Conclusions

SGLT2 inhibitors have shown unexpected clinical benefit regarding heart and kidney protection both within and outside the context of T2DM, suggesting potential intrinsic organ protective effects. This is supported by preclinical data that suggest a range of potential mechanisms of action, not limited to hemodynamic effects. These mechanisms of action may impact cell resistance to diverse stressors by decreasing oxidative stress and inflammation. Thus, SGLT2 inhibitors may be potentially beneficial as organ protective agents in COVID-19 (Table 1). However, given the potential risks of SGLT2 inhibitors in acutely ill patients, any use of these agents for COVID-19 patients with severe disease should be in the context of a clinical trial. Fortunately, results of the ongoing DARE-19 dapagliflozin in respiratory failure trial in patients with COVID-19 are expected by December 2020, thus potentially on time for second or third waves of COVID-19. Additionally, the results of this trial may guide the field of SGLT2 inhibitors in organ protection beyond viral infection or diabetes.

## Figures and Tables

**Table 1:** SGLT2 Inhibitors and COVID-19.

Current Status	Potential for the Future
Health agencies recommendation to avoid SGLT2 inhibitors during COVID-19 (“sick day rules”) Risk of volume depletion Hypotension Ketoacidosis Potential drug interactions (canagliflozin and lopinavir/ritonavir)	SGLT2 inhibitors and organ protection in diabetes and outside diabetes Clinical: heart failure, CKD Preclinical: lung Ongoing RCT to assess organ protection in COVID-19
